# The relationship between stress during pregnancy with leptin and cortisol blood concentrations and complications of pregnancy in the mother

**DOI:** 10.4274/jtgga.galenos.2019.2019.0010

**Published:** 2019-11-28

**Authors:** Soheila Rabiepour, Ehsan Saboory, Maryam Abedi

**Affiliations:** 1 Department of Midwifery & Reproductive Health Research Center, Urmia University of Medical Sciences, Urmia, Iran; 2 Islamic Azad University Faculty of Nursing and Midwifery Bonab, Miandoab, Iran

**Keywords:** Stress, cortisol, leptin, pregnancy complications

## Abstract

**Objective:**

Pregnancy is one of the most stressful periods a woman experiences in her life. The present study was an attempt to determine the relationship between maternal stress during pregnancy and cortisol plus maternal serum leptin concentrations as well as pregnancy outcomes.

**Material and Methods:**

This longitudinal study was conducted on 90 pregnant women in Miandoab city between 2015 and 2016. The samples were chosen from mothers with a gestational age of 24 to 28 weeks. The participants were asked to complete Cohen’s Perceived Stress Scale (PSS) and a demographic questionnaire and blood samples were taken from them. The mothers were then tracked with four-week intervals until the time of delivery and were asked to complete Cohen’s PSS each time along with a questionnaire related to maternal outcomes. Again, a blood sample was taken at the time of delivery. Data analysis was performed using SPSS 16. Descriptive statistics, Pearson’s correlation coefficient, and the t-test were employed for analysis.

**Results:**

A significant relationship was found between maternal stress and preeclampsia (p=0.008). The relationships between preterm childbirth and maternal cortisol concentrations in weeks 24-28 (p=0.015), and between preterm childbirth and maternal leptin concentrations at the time of delivery (p=0.007) were also found to be significant.

**Conclusion:**

Pregnancy and labor, as physically and mentally stressful events, can affect women’s physiologic and psychological indicators. As a consequence, during pregnancy, the cortisol and leptin index changes in response to the activity of the hypothalamic-pituitary axis and autonomic nervous system under stress.

## Introduction

Pregnancy is associated with significant physiologic and psychological changes, which sometimes lead to pathologic changes (1). Stressful situations can have adverse effects on women’s psychological status. Indeed, stress is the most obvious symptom in pregnant women’s behaviors and clinical symptoms (2). For instance, the prevalence of pregnancy stress in England and Sweden was reported as 33-37% and 5-7% respectively (3). Evidence suggests that mothers’ reaction to stress changes during pregnancy (4), which is directly correlated with pregnancy outcomes (5,6). Mothers’ stress also predicts delivery problems and complications (7). A pregnant woman’s stress affects her and leads to negative perceptions towards delivery and birth, unnecessary fear of childbirth and motherhood, self-medication with alcohol, and activity restriction (8). Studies suggest that preterm birth and gestational hypertension are more likely to occur in mothers with stressful pregnancies (9,10).

Pregnancy stress increases the chance of unplanned cesarean and labor complications (11,12). To measure the amount of stress during pregnancy, different methods such questionnaires (13) or measurement of biochemical markers, which are indicators of stress (14), are used. Today, cortisol and other hormones produced by the sympathetic nervous system activity, which can be measured from blood samples, are measured as indicators of stress (14). Cortisol, which is called the stress hormone, is considered as a decisive indicator in stressful situations (15,16). The hypothalamic-pituitary-adrenal axis is activated in response to stress and releases cortisol in the organism (17). The level of cortisol is a function of actual and perceived stress (18). In a study, Edwards et al. (19) examined the effects of stress during pregnancy on changes in hormonal and behavioral responses affecting serum concentrations of cortisol and leptin. They found that adipose tissue had an effect on the response to stress with secretion of leptin. Glucocorticoids and probably adrenocorticotropic hormone stimulate synthesis and secretion of leptin (20).

A number of studies have investigated the relationship between maternal stress and concentrations of cortisol, and have obtained contradictory results. On the other hand, no studies were found to have only investigated the relationship between perceived maternal stress during pregnancy plus labor and leptin concentrations. Accordingly, the present study examines the relationship between stress during pregnancy and leptin plus cortisol concentrations as well as complications of pregnancy in pregnant women.

## Material and Methods

The present study is a longitudinal study conducted between November 2015 and the end of April 2016 in Miandoab city. The study population consisted of pregnant women of 24 to 28 weeks’ gestation. The samples were chosen through available sampling. The sample size was calculated as 90 participants using the correlation coefficient formula a d based on the study conducted by Alavian et al. (21) and considering the correlation coefficient between stress and cortisol concentration (0.312), confidence level of 5%, and statistical power of 80%.

The criterion for inclusion in the study was not having any underlying disease during and before pregnancy. The required information was collected from the reference laboratory where all pregnant women of the city referred to for routine check-ups within weeks 24-28 of pregnancy. The reference laboratory was selected so that the samples would have the same conditions. This laboratory had lower costs because it was public, and all pregnant women from all over the town (including villages) were referred to this center.

The objectives and method of study were explained to the participants and their written consents were obtained. Cohen’s perceived stress scale (PSS) and a questionnaire developed by the researcher about the demographics of the participants were completed by the participants. Also, mothers’ blood samples taken for routine pregnancy tests at 24-28 weeks of pregnancy were used to test the leptin and cortisol concentrations of mothers’ blood.

Subsequent follow-up of the mothers in the study was performed three times with 4-week intervals at 28-32 weeks and 34-36 weeks, and at the time of delivery. The first and second follow-ups were performed by telephone with the PSS completed by the participants; for the third follow-up, they were asked to call the researcher, via the number they were provided with, in case they went to the delivery room having gone into labor. Also, according to the first day of the last period, the delivery time was estimated and the researcher was present in the delivery room at the time that was determined for the delivery of the participants, even if the participants did not call the researcher. Thus, the third follow-up was performed through presence in the delivery room. At the time of labor, again the PSS and a questionnaire related to maternal outcome were completed. In this stage, the mothers’ blood samples that were taken as a routine at the time of reception were used to test the leptin and cortisol concentrations of the mothers’ blood. Due to the circadian rhythm of cortisol and in order to minimize the impact of this rhythm on cortisol concentrations, the time of maternal blood sampling in the first stage was within 8-10 a.m, while in the second stage it depended on the time of delivery. The blood samples were centrifuged for 10 minutes at 2500 rpm and their serum samples were separated. Serum samples were placed in closed and coded Eppendorf tubes and were kept in -20 °C in the laboratory. All samples were analyzed under the same conditions (environmental, time, place, and analyst). To measure leptin and cortisol concentrations, Bio Vendor kits with sensitivity of 0.2 ng/mL and specificity of 100% for leptin, and DiaMetra kits with sensitivity of 2.44 ng/mL and specificity of 100% for cortisol were used, respectively.

### Tools

1. Cohen’s PSS; the PSS includes 14 expressions investigating the participants’ feelings and thoughts as well as their general perceived stress during the last month. The 14-item questionnaire including seven negative items and seven positive items was used here. The items were rated on a 5-point Likert scale ranging from ‘Almost Never’ to ‘Almost Always.’ Items 4, 5, 6, 7, 9, 10, and 13 were reverse coded. The lowest and highest scores were zero and 56, respectively (13,22). Cronbach’s alpha for American and Iranian populations was found by Ghorbani et al. (23) as 0.86 and 0.81, respectively. The questionnaire’s construct validity was established at 0.63 and was significant at p<0.05 (24).

2. Demographic questionnaire; this questionnaire was developed by the researcher and had three parts: demographic information (age, address, pregnant women and their husbands’ level of education, and job), information about fertility (gravidity, type of delivery, parity, abortion, the pregnancy planning, neonate’s sex, desirability of the neonate’s sex, mother’s interest in pregnancy), and maternal outcomes (preeclampsia, preterm delivery, dystocia, excessive bleeding in childbirth, and spotting during pregnancy).

### Data analysis

Data analysis was performed using the SPSS 16 software package. Further, the relationship between factors (p<0.05) was determined using Pearson’s correlation coefficient and the t-test.

## Results

In the present study, the average age of the mothers was 27.48 (range, 15-40) years. The general and reproductive characteristics of the sample are displayed in Table 1.

Some psychological issues and problems were also investigated in this study: 86.7% had planned pregnancy, the pregnancy in approximately 91.1% of the mothers was intended, 86.7% were happy with their child’s sex, and 91.1% were interested in their pregnancy. 

Maternal PSS scores at different time points of pregnancy, as well as cortisol and leptin concentrations (ng/mL) in mother are shown in Table 2.

According to Table 3, there was no significant relationship between maternal perceived stress during pregnancy and the time of delivery, the two conditions (yes or no) related to preterm birth, dystocia, spotting during pregnancy, and bleeding at the time of delivery. Although there was no significant difference between yes or no conditions, the PSS was higher in the yes state in almost all cases. There was a significant relationship between PSS scores at the time of delivery and preeclampsia (p=0.028) (Table 4).

Cortisol concentrations at the delivery were significantly higher with score of perceived stress in 24-28 weeks (p=0.019, r=0.246) and total stress score (p=0.046, r=0.211) and there were non-significant correlations between maternal cortisol concentrations and maternal leptin concentrations and PPS scores in pregnancy and at the time of delivery (Table 5).

At the time of delivery, the leptin concentrations showed a significant and negative relationship with preterm childbirth (p=0.007), i.e. the leptin concentrations were lower in preterm childbirth. However, the relationship between preterm child birth and cortisol concentrations in weeks 24-28 was significant and positive (p=0.015), but there were no significant relations between maternal leptin and cortisol concentrations and complications of pregnancy in the mother.

## Discussion

The findings revealed a significant relationship between perceived maternal stress at the time of delivery and preeclampsia. Shamsi et al. (25) evaluated the risk factors of preeclampsia in Pakistani women and reported a higher level of stress in women with preeclampsia. In another study, it was found that stress had a significant relationship with preeclampsia. Further, stress had a significant relationship with the severity and worsening of preeclampsia (26). This is also confirmed by the findings of Black who suggested that women with severe preeclampsia had higher stress concentrations compared with those with a mild preeclampsia (27). The findings of the present study in this area agree with those of previous research.

A significant relationship was found here between cortisol concentrations at the time of delivery and perceived stress scores. Similarly, the relationship between perceived stress and cortisol concentrations in late pregnancy was found to be significant in another study, though no significant relationship was established between cortisol concentrations and early pregnancy (28). On the other hand, the findings revealed no significant relationship between perceived stress scores during pregnancy and plasma cortisol concentrations (29). A significant relationship was reported between severe stress based on visual analogue scale at the time of labor and salivary cortisol (21). In addition, Pluess et al. (30) reported a significant relationship between mothers’ state anxiety and salivary cortisol concentrations in early and late pregnancy. The findings of our study are in agreement with most previous studies in this regard. The difference between our findings and some other studies can be attributed to the use of different instruments to measure stress concentrations during pregnancy.

We found a significant relationship between preterm childbirth and maternal plasma cortisol concentrations in weeks 24-28 of pregnancy. Previous studies suggested that the mean concentrations of maternal plasma cortisol in women with preterm labor were higher compared with their counterparts. It indicates that cortisol plays a significant role in the mechanism of preterm labor in some women (31). In another study, plasma cortisol concentrations in women giving preterm birth were found higher than in cases of normal delivery, implying the importance of maternal hypercortisolemia in preterm labor (32). According to these findings, the risk of preterm delivery grows with high blood cortisol concentrations (31,33,34).

In the present study, a negative significant relationship was observed between preterm childbirth and maternal plasma leptin at the time of delivery. The mechanism of leptin in preterm birth is generally unknown in the literature. Some studies claim that increased concentrations of leptin in preterm delivery are closely linked to antenatal exposure to corticosteroids (35).

The literature suggests that the risk of preterm labor before week-34 of gestation decreases with higher concentrations of leptin. Wuntakal et al. (36) reported that induced myometrium contraction was determined by the availability of leptin and might prove helpful in preventing preterm birth. In a study on 1304 pregnant women in weeks 16-27 of gestation, Palchevska et al. (37) showed that the amount of maternal leptin was higher in women who delivered at term than in those with premature delivery. The difference was still observed after controlling for diabetes, blood pressure disorders, and pre-pregnancy body mass index. In the same vein, Palchevska et al. (37) studied 110 neonates and found that that leptin concentrations were higher for term infants. This was further confirmed in the study by Laivuori et al. (38).

### Study limitations

Environmental conditions and circadian rhythm affect cortisol concentrations. This was controlled, as much as possible, by taking samples in the morning in weeks 24-28 of pregnancy. However, at the time of delivery, due to its unexpected and emergency nature, it was beyond the researcher’s ability to control this condition.

The present study found a significant relationship between preeclampsia and average stress scores in pregnancy. In addition, there was a significant relationship between leptin and cortisol concentrations in maternal serum and preterm childbirth. These findings indicate the negative and undesirable impact of stress on pregnancy outcomes. Other studies can be conducted to discover the possibility of predicting pregnancy outcomes by measuring cortisol and leptin concentrations in blood serums at other stages of pregnancy. Note that in order to draw safer conclusions, there is a need for more longitudinal studies with larger samples.

**Ethical Issues:** Information about the participants remained confidential thought the study and the results were disseminated collectively.

## Figures and Tables

**Table 1:**
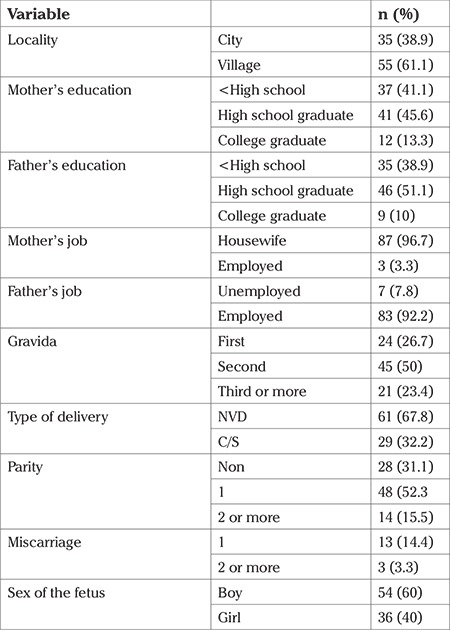
General and reproductive characteristics of the sample

**Table 2:**
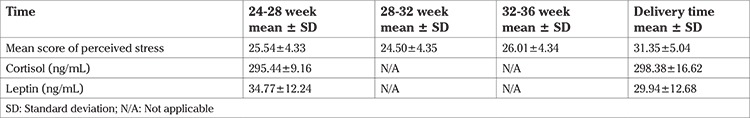
Maternal perceived stress score and cortisol and leptin concentrations (ng/mL) of the sample (mean ± standard deviation)

**Table 3:**
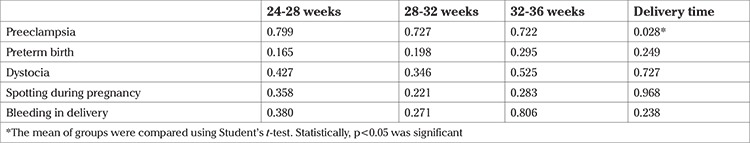
The relationship between perceived mother’s stress score and complications of pregnancy (p value)

**Table 4:**
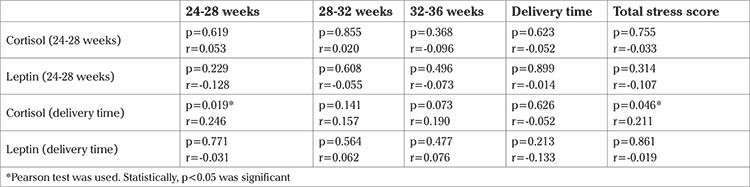
The relationship between maternal perceived stress and cortisol and leptin concentrations of the mother

**Table 5:**
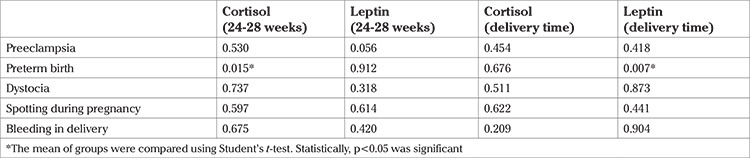
The relationship between maternal cortisol and leptin with complications of pregnancy (p value)
